# Association of Bowel Urgency With Quality-of-Life Measures in Patients With Moderately-to-Severely Active Ulcerative Colitis: Results From Phase 3 LUCENT-1 (Induction) and LUCENT-2 (Maintenance) Studies

**DOI:** 10.1093/crocol/otae001

**Published:** 2024-01-06

**Authors:** Millie D Long, Stefan Schreiber, Toshifumi Hibi, Theresa Hunter Gibble, Deborah A Fisher, Gina Park, Richard E Moses, Peter D R Higgins, James O Lindsay, Scott D Lee, Rodrigo Escobar, Vipul Jairath

**Affiliations:** Division of Gastroenterology and Hepatology, University of North Carolina, Chapel Hill, NC, USA; Department Internal Medicine I, University Hospital Schleswig-Holstein, Kiel University, Kiel, Germany; Kitasato Institute Hospital, Minato-ku, Center for Advanced IBD Research and Treatment, Tokyo, Japan; Eli Lilly and Company, Indianapolis, IN, USA; Eli Lilly and Company, Indianapolis, IN, USA; Techdata Services Company LLC, King of Prussia, PA, USA; Eli Lilly and Company, Indianapolis, IN, USA; Department of Medicine, Division of Gastroenterology, University of Michigan, Ann Arbor, MI, USA; Barts Health NHS Trust, London, UK; Division of Gastroenterology, University of Washington Medical Center, Seattle, WA, USA; Eli Lilly and Company, Indianapolis, IN, USA; Department of Medicine, Division of Gastroenterology, Western University, London, Ontario, Canada

**Keywords:** bowel urgency, ulcerative colitis, quality of life

## Abstract

**Background:**

Improvement in bowel urgency (BU) was associated with better clinical outcomes in phase 3 LUCENT-1 (induction) and LUCENT-2 (maintenance) studies in moderately-to-severely active ulcerative colitis (UC). We assessed association of BU with quality-of-life (QoL) outcomes.

**Methods:**

LUCENT-1: 1162 patients randomized 3:1 to intravenous mirikizumab 300 mg or placebo every 4 weeks (Q4W) for 12 weeks. LUCENT-2: 544 mirikizumab induction responders re-randomized 2:1 to subcutaneous mirikizumab 200 mg or placebo Q4W through Week (W) 40 (W52 of continuous treatment). Patients reported BU severity in the past 24 hours using a validated Urgency Numeric Rating Scale (NRS). In patients with baseline Urgency NRS ≥3, the association between BU Clinically Meaningful Improvement (CMI; ≥3-point decrease) and remission (score 0 or 1) with patient-reported outcomes was assessed at W12 and W52.

**Results:**

A significantly greater proportion of patients with versus without BU Remission achieved IBDQ remission (W12: 87.3% vs 42.7%, *P* < .0001; W52: 91.4% vs 45.5%, p < .0001). Similarly, BU Remission was associated with more patients achieving CMI in SF-36 Physical Component Summary (W12: 69.0% vs 44.4%, *P* < .0001; W52: 77.5% vs 42.1%, *P* < .0001) and Mental Component Summary (W12: 53.5% vs 41.0%, *P* = .0019; W52: 62.0% vs 38.3%, *P* < .0001) scores. At W12 and W52, patients with BU CMI or Remission showed significant improvements in EQ-5D-5L and Work Productivity and Activity Impairment:UC scores. Significant improvements were also seen in fatigue, abdominal pain, and nocturnal stool.

**Conclusions:**

In patients with moderately-to-severely active UC, improvement in BU was associated with improved QoL in phase 3 LUCENT-1 and LUCENT-2 studies.

**Clinical Studies:**

LUCENT-1: NCT03518086; LUCENT-2: NCT03524092

## Graphical Abstract

**Graphical Abstract:**
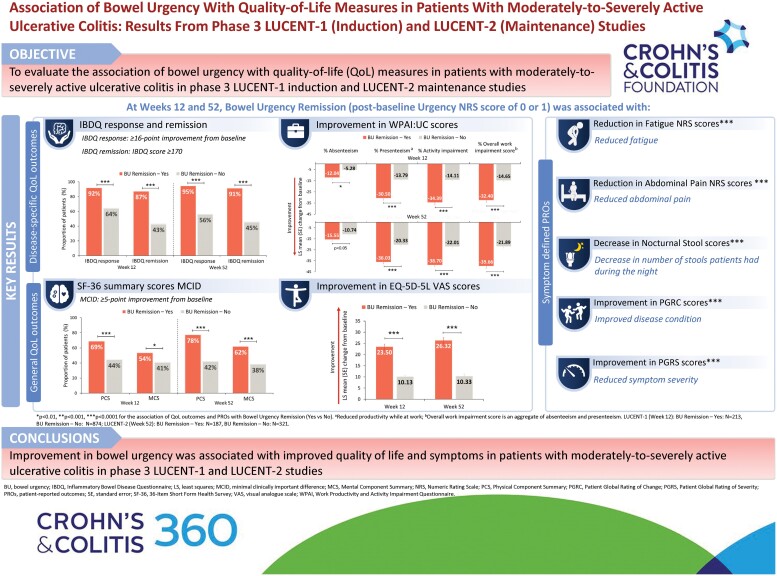

## Introduction

Ulcerative colitis (UC) is a chronic idiopathic inflammatory bowel disease (IBD) of complex etiology, affecting genetically susceptible individuals.^1,2^ Typical symptoms of the disease include diarrhea, rectal bleeding (RB), bowel urgency, and abdominal pain.^1,2^ These symptoms can result in impaired quality of life (QoL) across psychological, physical, and social domains.^2-4^

Bowel urgency, the sudden or immediate need to have a bowel movement, is one of the most aggravating symptoms and is experienced by more than 80% of patients with UC.^5,6^ Bowel urgency is often associated with symptoms that can be reflective of active UC. However, some patients report bowel urgency even in the absence of symptoms that usually indicate active mucosal inflammation.^7^

Several studies have reported that bowel urgency has a negative impact on patients’ QoL.^8-10^ Bowel urgency has been shown to be an independent symptom separate from increased stool frequency (SF) and RB.^10-15^ In previous treatment-agnostic mediation analyses including combined patients from mirikizumab and placebo groups from LUCENT-1 and LUCENT-2 trials, relative to RB remission and SF remission, Bowel Urgency Remission and Bowel Urgency Clinically Meaningful Improvement (CMI) primarily accounted for improvements in Inflammatory Bowel Disease Questionnaire (IBDQ) scores,^11^ Patient Global Rating of Severity (PGRS),^12^ Patient Global Rating of Change (PGRC),^12^ Work Productivity and Activity Impairment (WPAI) scores,^13^ and fatigue.^14^ Results from upadacitinib phase 3 induction studies also showed that bowel urgency largely mediated improvement in endoscopic and histologic endpoints.^15^ In a phase 2 study, significantly higher rates of achieving SF remission, RB remission, and endoscopic healing were observed in patients who reported absence of urgency.^10^ However, historically, bowel urgency was not endorsed as an endpoint in clinical trials,^16^ and when included, it was evaluated only as a binary (yes vs no) assessment.^17^

Mirikizumab (LY3074828) is a humanized immunoglobulin G4 monoclonal antibody that inhibits interleukin (IL)-23 by binding to an epitope on the p19 subunit. In the phase 2 study (NCT02589665) of mirikizumab in patients with moderately-to-severely active UC, absence of bowel urgency was strongly associated with improvement in QoL and clinical measures of UC disease activity.^10^ In the phase 3 LUCENT-1 and LUCENT-2 studies in patients with moderately-to-severely active UC, patients who achieved Bowel Urgency CMI or Remission also reported significantly better clinical outcomes and normalized fecal calprotectin and C-reactive protein.^18^

The current study aims to assess the association of bowel urgency with QoL measures in the LUCENT-1 and LUCENT-2 studies.

## Methods

### Study Design and Population

Data from LUCENT-1 (12-week induction; NCT03518086) and LUCENT-2 (40-week maintenance; NCT03524092) phase 3, multicenter, randomized, double-blind, parallel-arm, placebo-controlled studies in patients with moderately-to-severely active UC were analyzed. The study design, eligibility criteria, methodology, and statistical analysis of these studies are published elsewhere.^19^

Briefly, adult patients with moderately-to-severely active UC at screening (modified Mayo score [MMS] of 4-9 with an endoscopic subscore ≥2 as assessed by blinded central reading^19^) who had failed (inadequate response, loss of response, or intolerance) conventional therapy (corticosteroid or immunomodulator) or advanced therapy (biologics or Janus kinase inhibitors) were included. Patients were excluded if they had received anti-IL12/23p40 antibodies or anti-IL-23p19 antibodies or had failed ≥3 biologic therapies for UC.

Study protocols and informed consent forms were approved by the ethical review board supervising each site. The studies were compliant with International Conference on Harmonization Good Clinical Practice guidelines, Declaration of Helsinki, and Council for International Organizations of Medical Sciences International Ethical Guidelines. Written informed consent was provided by all patients.

### Randomization and Treatments

Patients in the LUCENT-1 study were randomized 3:1 to receive mirikizumab 300 mg or placebo intravenously every 4 weeks (Q4W) up to Week 12. At Week 12 (LUCENT-1), mirikizumab induction responders (patients who achieved ≥2 points and ≥30% decrease from baseline in MMS, and ≥1 point decrease from baseline in the RB subscore or a RB score of 0 or 1) were re-randomized 2:1 to receive mirikizumab 200 mg or placebo subcutaneously Q4W up to Week 40 in the LUCENT-2 study. LUCENT-1 and LUCENT-2 together constituted a total of 52 weeks of continuous therapy, with Week 12 representing the end of induction study and the beginning (Week 0) of the 40-week maintenance study.

### Study Outcomes and Assessments

#### Bowel urgency

The urgency numeric rating scale (NRS) is a validated, single patient-reported item that measures bowel urgency severity.^20^ Patients were asked to rate the severity of urgency to have a bowel movement in the past 24 hours using an 11-point NRS ranging from 0 (no urgency) to 10 (worst possible urgency).^21^ Patients recorded bowel urgency severity daily using an eDiary; weekly scores were calculated for each patient using an average of each 7-day period rounded to the nearest integer. Missing data for weekly assessments was defined as data recorded for fewer than 4 days.

Bowel Urgency CMI was defined as ≥3-point improvement from baseline, and Bowel Urgency Remission was defined as achieving a post-baseline score of 0 or 1 (minimal to no urgency) in patients with baseline Urgency NRS ≥3. These thresholds were defined based on psychometric analyses,^20^ qualitative interviews,^21^ and anchor-based analyses conducted using PGRC, PGRS, and clinical remission as anchor variables.^22^

#### Patient-reported outcome (PRO) measures

Detailed descriptions of PRO measures are provided in Table S1.

Patient-reported QoL outcomes included IBDQ (total scores: 32-224),^23-25^ Medical Outcomes Study 36-Item Short Form Health Survey (SF-36; version 2),^26,27^ EQ-5D-5L,^28,29^ and WPAI:UC.^30,31^ For all QoL outcomes, patient responses were recorded electronically on a tablet device at respective timepoints.

PROs included Fatigue NRS, Abdominal Pain NRS, PGRS, PGRC, and Nocturnal Stool. Fatigue NRS and Abdominal Pain NRS are single patient-reported items measured on an 11-point NRS ranging from 0 (no fatigue/no abdominal pain) to 10 (fatigue as bad as can imagine/worst possible abdominal pain) in the past 24 hours. PGRS, a single-item questionnaire, was used to assess patients’ rating of disease symptom severity over the past 24 hours on a 6-point scale (1 = “no symptoms” and 6 = “very severe”). PGRC assessed patients’ rating of change in their symptom(s) on a 7-point Likert scale (1 = “very much better,” 4 = “no change,” and 7 = “very much worse”). Nocturnal Stool, a single-item instrument, was used to record the number of stools patients had during the night (or day, for shift workers) causing them to wake from sleep. Responses were collected electronically at respective timepoints using an eDiary (Fatigue NRS, Abdominal Pain NRS, Nocturnal Stool, and PGRS) or tablet (PGRC); weekly scores (except PGRC) were calculated for each patient using an average of each 7-day period rounded to the nearest integer.

#### Association of bowel urgency with PRO measures

Associations of 'Bowel Urgency CMI' or 'Bowel Urgency Remission' with (1) change from baseline in IBDQ, SF-36, EQ-5D-5L, and WPAI:UC, (2) IBDQ response (≥16-point improvement from baseline)^23^ and IBDQ remission (IBDQ score ≥170),^32^ and (3) SF-36 Physical and Mental Component Summary (PCS and MCS, respectively) scores minimal clinically important difference (MCID; ≥5-point improvement from baseline)^33^ were evaluated at Weeks 12 and 52.

Associations of 'Bowel Urgency CMI' and 'Bowel Urgency Remission' with change from baseline in (1) Fatigue NRS, (2) Abdominal Pain NRS, (3) PGRS, (4) PGRC, and (5) Nocturnal Stool were also assessed at Weeks 12 and 52.

### Statistical Analyses

The statistical analyses were performed using SAS Version 9.4.

The modified intent-to-treat (mITT) population included all randomized patients who received any amount of study treatment excluding patients impacted by the electronic clinical outcome assessment transcription error in the wording used for the assessment of RB (Poland) and SF (Turkey) Mayo subscores.^19^ Patients in the mITT population who had baseline Urgency NRS ≥3 were included in the induction study (LUCENT-1) analyses; patients from mirikizumab and placebo treatment groups were combined for the analyses. For the maintenance study (LUCENT-2) analyses, patients who had Urgency NRS ≥3 at LUCENT-1 baseline and achieved clinical response with mirikizumab therapy at Week 12 (LUCENT-1) were included. Similar to the induction study analyses, patients from LUCENT-2 mirikizumab and placebo treatment groups were combined for the maintenance study analyses.

Patient demographics and baseline characteristics were summarized for the mITT population with Urgency NRS ≥3 at baseline. Descriptive statistics were used to summarize continuous variables, and frequency counts and percentages were used to summarize categorical variables.

Baseline for induction and maintenance studies was defined as the baseline of induction treatment (the last non-missing assessment recorded on or prior to the date of the first study drug administration at Week 0). Change from baseline in continuous PRO measures was analyzed using an analysis of covariance model that included baseline, treatment group, and bowel urgency response (or remission). Least squares mean (LSM), LSM difference, and *P*-value were reported. Missing data were imputed using a modified baseline observation carried forward method.

Cochran–Mantel–Haenszel test, adjusted for the treatment group, was used to compare the proportion of patients who achieved MCID (SF-36 PCS and MCS MCID response rates, and IBDQ response and IBDQ remission rates) in the Bowel Urgency CMI and Bowel Urgency Remission status groups at Weeks 12 and 52. Pooled odds ratio with 95% CI and *P*-value were reported. Missing data were imputed using non-responder imputation.

## Results

### Patient Population

In the induction study analyses, 1087 patients with baseline Urgency NRS ≥3 were included. At Week 12, Bowel Urgency CMI (Urgency NRS ≥3 improvement) was observed in 484 patients (44.5%) and was absent in 603 patients (55.5%). Patient demographics and baseline characteristics were generally balanced between the 2 groups (Table 1). A total of 213 (19.6%) out of 1087 patients achieved Bowel Urgency Remission (Urgency NRS = 0 or 1) at Week 12.

**Table 1.** Demographics and baseline disease characteristics (mITT population with Urgency NRS score ≥3 at baseline by Urgency NRS ≥3 improvement [CMI] status at Week 12 [LUCENT-1], NRI).

		Bowel Urgency CMI at Week 12 (LUCENT-1)	
		Bowel Urgency CMI − Yes (*N* = 484)	Bowel Urgency CMI − No (*N* = 603)
Age (years), mean (SD)		41.9 (13.49)	42.7 (13.99)
Male, *n* (%)		291 (60.1%)	356 (59.0%)
BMI (kg/m^2^), mean (SD)		24.79 (5.208)	24.89 (5.389)
Prior biologic or tofacitinib failure, *n* (%)		179 (37.0%)	280 (46.4%)
Baseline UC therapy, n (%)	Corticosteroid	202 (41.7%)	235 (39.0%)
	Immunomodulator	110 (22.7%)	152 (25.2%)
Duration of UC (years), mean (SD)		6.90 (6.983)	7.35 (6.743)
Baseline disease location, *n* (%)	Proctitis	2 (0.4)	6 (1.0)
	Left-sided colitis	322 (66.5)	363 (60.3)
	Pancolitis	160 (33.1)	233 (38.7)
Modified Mayo score, mean (SD)		6.8 (1.23)	6.4 (1.30)
Modified Mayo score category, *n* (%)	Moderate (4-6)	188 (38.8%)	292 (48.5%)
	Severe (7-9)	296 (61.2%)	309 (51.3%)
Total Mayo score, mean (SD)		9.1 (1.51)	8.7 (1.56)
Total Mayo score category, *n* (%)	Moderate (6-9)	267 (57.7%)	379 (66.1%)
	Severe (10-12)	192 (41.5%)	190 (33.2%)
Baseline Urgency NRS, mean (SD)		6.9 (1.58)	6.2 (1.98)
IBDQ total score^a^ (range: 32-224), mean (SD)		126.3 (32.52)	129.5 (32.44)
IBDQ subscores^a^, mean (SD)	Bowel symptoms (range: 10-70)	36.9 (9.40)	38.6 (10.09)
	Systemic symptoms (range: 5-35)	17.9 (5.51)	18.4 (5.54)
	Emotional function (range: 12-84)	51.6 (14.23)	52.1 (13.83)
	Social function (range: 5-35)	19.9 (7.35)	20.4 (7.59)
SF-36 MCS^a^ (range: 0-100), mean (SD)		43.3 (10.04)	43.4 (10.32)
SF-36 PCS^a^ (range: 0-100), mean (SD)		41.3 (7.77)	42.0 (7.93)
EQ-5D-5L VAS^a^ (range: 0-100), mean (SD)		54.0 (18.66)	55.0 (19.69)
Employment status (Yes), *n* (%)		293 (60.9%)	366 (61.7%)
Overall WPAI:UC score^b^, mean (SD)		50.4 (25.81)	50.1 (25.74)
Abdominal Pain NRS (range: 0-10)^c^, mean (SD)		5.7 (2.23)	4.9 (2.34)
Abdominal Pain NRS, *n* (%)	<4	76 (15.7%)	174 (28.9%)
	≥4	408 (84.3%)	429 (71.1%)
Fatigue NRS^c^ (range: 0-10), mean (SD)		6.3 (2.04)	5.7 (2.08)
PGRS^d^ (range: 1-6), mean (SD)		4.5 (0.75)	4.2 (0.75)
Nocturnal stool^e^, mean (SD)		2.0 (3.36)	2.2 (4.24)

^a^Higher score indicates better quality of life/health.

^b^Overall work impairment score is an aggregate of absenteeism and presenteeism.

^c^Score 0 = “no abdominal pain/no fatigue” and 10 = “worst possible abdominal pain/fatigue as bad as can imagine”.

^d^Score 1 = “no symptoms” and 6 = “very severe”.

^e^Number of stools patients had during the night (or day, for shift workers) causing them to wake from sleep.

BMI, body mass index; CMI, Clinically Meaningful Improvement; IBDQ, Inflammatory Bowel Disease Questionnaire; MCS, Mental Component Summary; mITT, modified intent-to-treat population; NRI, non-responder imputation; NRS, numeric rating scale; PCS, Physical Component Summary; PGRS, Patient’s Global Rating of Severity; SD, standard deviation; SF-36, Medical Outcomes Study 36-Item Short Form Health Survey; UC, ulcerative colitis; VAS, visual analog scale; WPAI:UC, Work Productivity and Activity Impairment Questionnaire:Ulcerative colitis.

In the maintenance study analyses, 508 patients with baseline Urgency NRS ≥ 3 who achieved clinical response with mirikizumab therapy at Week 12 were included. Of these, 291 (57.3%) patients achieved Bowel Urgency CMI, and 187 (36.8%) patients achieved Bowel Urgency Remission at Week 52.

### Association Between Bowel Urgency CMI or Bowel Urgency Remission Status and Patient-Reported Outcomes

#### Inflammatory Bowel Disease Questionnaire

**Figure 1.**
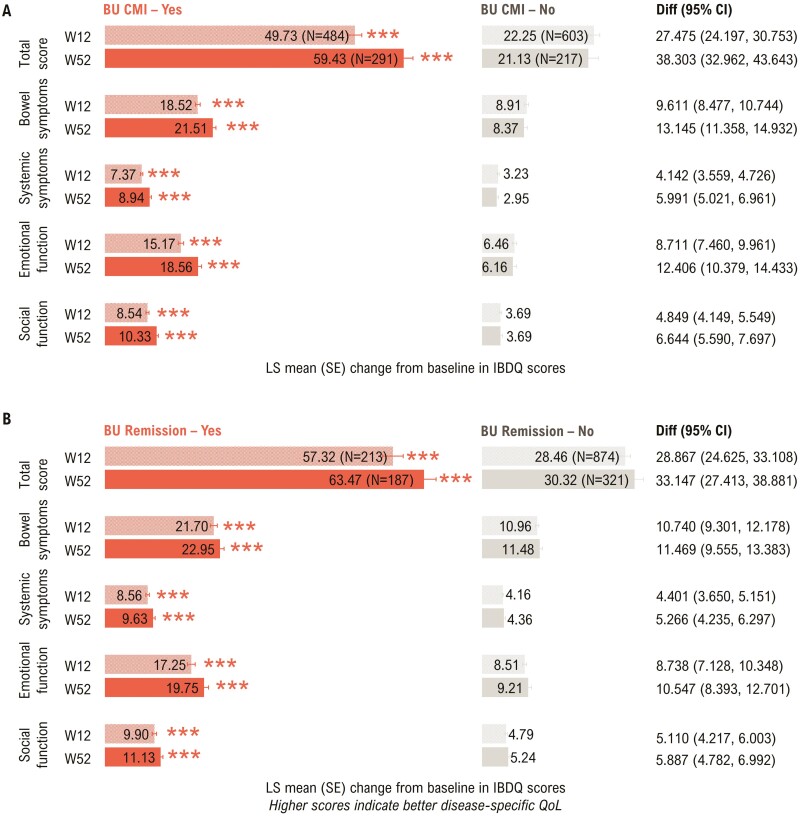
Association between Bowel Urgency CMI or Remission and IBDQ total and domain scores at Weeks 12 and 52 − mITT with baseline Urgency NRS ≥3^a^. *P*-values were calculated using ANCOVA with mBOCF for missing data imputation. ***P < .0001 for the association of IBDQ outcomes with Bowel Urgency CMI or Remission (Yes vs No). ^a^Induction study analyses included mITT patients with baseline Urgency NRS ≥3. Maintenance study analyses included patients who had Urgency NRS ≥3 at baseline and achieved clinical response with mirikizumab at Week 12 (LUCENT-1). ANCOVA, analysis of covariance; BU, bowel urgency; CI, confidence interval; CMI, Clinically Meaningful Improvement; Diff, difference; IBDQ, Inflammatory Bowel Disease Questionnaire; LS, least squares; mBOCF, modified baseline observation carried forward; mITT, modified intent-to-treat; NRS, numeric rating scale; QoL, quality of life; SE, standard error; W, week.

Patients achieving Bowel Urgency CMI or Bowel Urgency Remission showed significantly higher (*P* < .0001) improvements in IBDQ total and domain scores than those who did not achieve Bowel Urgency CMI or Bowel Urgency Remission (Figure 1A–B). Patients achieving Bowel Urgency CMI had significantly higher IBDQ response rates at Week 12 (88.6% vs 54.4%; *P* < .0001) and Week 52 (92.4% vs 40.6%; *P* < .0001, Figure S1A). Similarly, patients achieving Bowel Urgency Remission showed significantly higher IBDQ response rates at Week 12 (92.0% vs 64.2%; *P* < .0001) and Week 52 (94.7% vs 56.1%; *P* < .0001, Figure S1A).

A significantly higher proportion of patients achieving Bowel Urgency CMI reported IBDQ remission at Week 12 (69.0% vs 37.3%; *P* < .0001) and Week 52 (83.8% vs 33.6%; *P* < .0001, Figure S1B). Similarly, patients achieving Bowel Urgency Remission had significantly higher IBDQ remission rates at Week 12 (87.3% vs 42.7%; *P* < .0001) and Week 52 (91.4% vs 45.5%; *P* < .0001, Figure S1B).

#### Medical outcomes study 36-item short-form health survey

**Figure 2.**
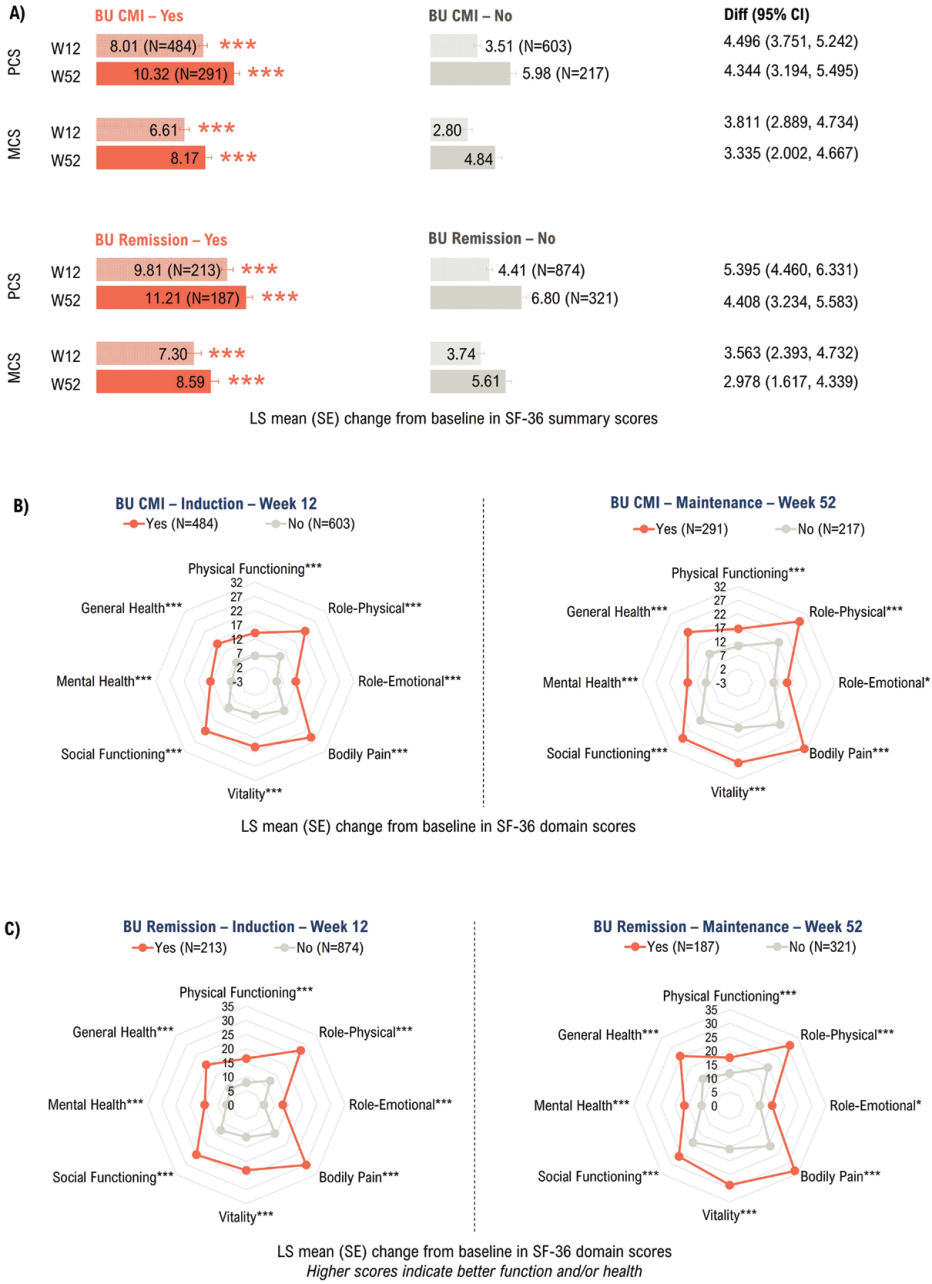
Association between Bowel Urgency CMI or Remission and SF-36 summary scores (A) and domain scores (B and C) at Week 12 and Week 52—mITT with baseline Urgency NRS ≥3^a^. *P*-values were calculated using ANCOVA with mBOCF for missing data imputation. *P < .01, **P < .001, ***P < .0001 for the association of SF-36 outcomes with Bowel Urgency CMI or Remission (Yes vs No). ^a^Induction study analyses included mITT patients with baseline Urgency NRS ≥ 3. Maintenance study analyses included patients who had Urgency NRS ≥ 3 at baseline and achieved clinical response with mirikizumab at Week 12 (LUCENT-1). ANCOVA, analysis of covariance; BU, bowel urgency; CI, confidence interval; CMI, Clinically Meaningful Improvement; Diff, difference; LS, least squares; mBOCF, modified baseline observation carried forward; MCS, Mental Component Summary; mITT, modified intent-to-treat; NRS, Numeric Rating Scale; PCS, Physical Component Summary; SE, standard error; SF-36, 36-Item Short Form Health Survey; W, week.

Bowel Urgency CMI and Bowel Urgency Remission were associated with significant improvements in SF-36 PCS and MCS scores (*P* < .0001; Figure 2A), and domain scores (*P* < .01 for Bowel Urgency CMI; *P* < .01 for Bowel Urgency Remission; Figure 2B and 2C) at Weeks 12 and 52.

In patients with Bowel Urgency CMI, PCS MCID response rates were significantly higher at Week 12 (64.3% vs 37.2%; *P* < .0001) and Week 52 (74.9% vs 28.6%; *P* < .0001, Figure S2A). Likewise, Bowel Urgency Remission was associated with significantly improved PCS MCID response rates at Week 12 (69.0% vs 44.4%; *P* < .0001) and Week 52 (77.5% vs 42.1%; *P* < .0001, Figure S2B).

Patients who achieved Bowel Urgency CMI had significantly higher MCS MCID response rates at Weeks 12 (53.9% vs 35.0%; *P* < .0001) and 52 (62.5% vs 26.3%; *P* < .0001, Figure S2A). Similarly, patients achieving Bowel Urgency Remission showed significant improvement in MCS MCID response rates at Weeks 12 (53.5% vs 41.0%; *P* = .0019) and 52 (62.0% vs 38.3%; *P* < .0001, Figure S2B).

#### EQ-5D-5L

**Figure 3.**
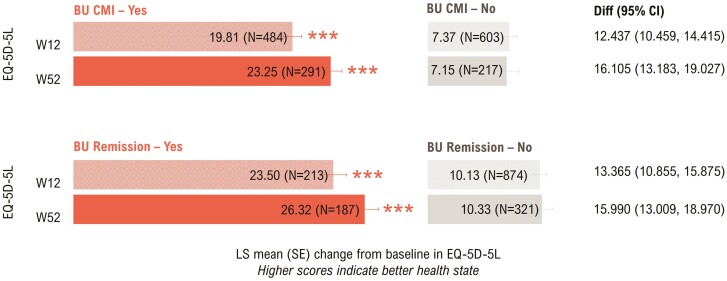
Association between Bowel Urgency CMI or Remission and EQ-5D-5L visual analog scale at Weeks 12 and 52—mITT with baseline Urgency NRS ≥3^a^. *P*-values were calculated using ANCOVA with mBOCF for missing data imputation. ***P < .0001 for the association of EQ-5D-5L outcomes with Bowel Urgency CMI or Remission (Yes vs No). ^a^Induction study analyses included mITT patients with baseline Urgency NRS ≥3. Maintenance study analyses included patients who had Urgency NRS ≥3 at baseline and achieved clinical response with mirikizumab at Week 12 (LUCENT-1). ANCOVA, analysis of covariance; BU, bowel urgency; CI, confidence interval; CMI, Clinically Meaningful Improvement; Diff, difference; LSM, least squares mean; mBOCF, modified baseline observation carried forward; mITT, modified intent-to-treat; NRS, Numeric Rating Scale; SE, standard error.

Bowel urgency CMI and Bowel Urgency Remission were associated with significant improvement in EQ-5D-5L visual analog scale (VAS) scores at Weeks 12 and 52 (*P* < .0001, Figure 3).

#### Work Productivity and Activity Impairment Questionnaire: UC

Patients achieving Bowel Urgency CMI showed significant decrease in all 4 scores at Week 12: absenteeism (*P* = .0004); presenteeism, activity impairment, and overall work impairment (all *P* < .0001, Table 2). At Week 52, in patients achieving Bowel Urgency CMI, significant decrease was sustained for 3 of the 4 domain scores: presenteeism, activity impairment, and overall work impairment (*P* < .0001) (Table 2).

**Table 2.** Association between Bowel Urgency CMI or Remission and change from baseline in WPAI:UC, Abdominal Pain NRS, and Fatigue NRS at Week 12 and Week 52 − mITT with baseline Urgency NRS ≥3.^a^

BU CMI				
LSM (SE) change from baseline in QoL measures	BU CMI − Week 12		LSM difference (95% CI)	*P*-value
	Yes (*N* = 484)	No (*N* = 603)		
WPAI:UC^b^				
% Absenteeism	–10.18 (1.443)	–3.94 (1.256)	–6.245 (–9.719, –2.772)	.0004
% Presenteeism^c^	–25.66 (1.350)	–10.90 (1.180)	–14.764 (–18.010, –11.519)	<.0001
% Activity impairment	–28.52 (1.317)	–10.29 (1.125)	–18.228 (–21.376, –15.080)	<.0001
% Overall work impairment score^d^	–26.48 (1.538)	–12.14 (1.345)	–14.343 (–18.041, –10.644)	<.0001
Fatigue NRS	–3.16 (0.095)	–0.73 (0.080)	–2.435 (–2.661, –2.208)	<.0001
Abdominal Pain NRS	–3.46 (0.087)	–1.00 (0.073)	–2.460 (–2.668, –2.252)	<.0001

LSM (SE) change from baseline in QoL measures	BU CMI − Week 52		LSM difference (95% CI)	*P*-value
	Yes (*N* = 291)	No (*N* = 217)		
WPAI:UC^b^				
% Absenteeism	–13.87 (1.364)	–10.59 (1.453)	–3.279 (–7.164, 0.606)	.0978
% Presenteeism^c^	–32.92 (1.476)	–17.26 (1.611)	–15.654 (–19.931, –11.377)	<.0001
% Activity impairment	–35.11 (1.513)	–18.76 (1.646)	–16.345 (–20.714, –11.975)	<.0001
% Overall work impairment score^d^	–35.03 (1.717)	–19.77 (1.873)	–15.258 (–20.222, –10.294)	<.0001
Fatigue NRS	–3.73 (0.132)	–1.06 (0.142)	–2.668 (–3.045, –2.291)	<.0001
Abdominal Pain NRS	–4.07 (0.113)	–1.33 (0.122)	–2.740 (–3.064, –2.415)	<.0001

BU remission				
LSM (SE) change from baseline in QoL measures	BU remission − Week 12		LSM difference (95% CI)	*P*-value
	Yes (*N* = 213)	No (*N* = 874)		
WPAI:UC^b^				
% Absenteeism	–12.04 (2.020)	–5.28 (1.093)	–6.755 (–10.979, –2.531)	.0018
% Presenteeism^c^	–30.50 (1.867)	–13.79 (1.041)	–16.716 (–20.643, –12.789)	<.0001
% Activity impairment	–34.39 (1.906)	–14.11 (1.000)	–20.282 (–24.256, –16.307)	<.0001
% Overall work impairment score^d^	–32.40 (2.109)	–14.65 (1.173)	–17.753 (–22.193, –13.314)	<.0001
Fatigue NRS	–3.85 (0.144)	–1.27 (0.073)	–2.576 (–2.877, –2.276)	<.0001
Abdominal Pain NRS	–4.12 (0.133)	–1.55 (0.068)	–2.573 (–2.850, –2.296)	<.0001

LSM (SE) change from baseline in QoL measures	BU remission − Week 52		LSM difference (95% CI)	*P*-value
	Yes (*N* = 187)	No (*N* = 321)		
WPAI:UC^b^				
% Absenteeism	–15.55 (1.704)	–10.74 (1.210)	–4.813 (–8.872, –0.755)	.0203
% Presenteeism^c^	–36.03 (1.830)	–20.33 (1.347)	–15.702 (–20.114, –11.289)	<.0001
% Activity impairment	–38.70 (1.894)	–22.01 (1.364)	–16.695 (–21.221, –12.169)	<.0001
% Overall work impairment score^d^	–39.66 (2.084)	–21.89 (1.535)	–17.765 (–22.794, –12.736)	<.0001
Fatigue NRS	–4.19 (0.163)	–1.61 (0.120)	–2.581 (–2.970, –2.192)	<.0001
Abdominal Pain NRS	–4.52 (0.142)	–1.90 (0.104)	–2.629 (–2.966, –2.291)	<.0001

Data are presented as LSM (SE) change from baseline. *P*-values were calculated using ANCOVA with mBOCF for missing data imputation. Higher negative values indicate improvement.

^a^Induction study analyses included mITT patients with baseline Urgency NRS ≥3. Maintenance study analyses included patients who had Urgency NRS ≥3 at baseline and achieved clinical response with mirikizumab at Week 12 (LUCENT-1).

^b^For WPAI:UC, mITT patients with baseline employment status “yes” were included.

^c^Reduced productivity while at work.

^d^Overall work impairment score is an aggregate of absenteeism and presenteeism.

ANCOVA, analysis of covariance; BU, bowel urgency; CI, confidence interval; CMI, Clinically Meaningful Improvement; LSM, least squares mean; mBOCF, modified baseline observation carried forward; mITT, modified intent-to-treat; NRS, Numeric Rating Scale; QoL, quality of life; SE, standard error; WPAI:UC, Work Productivity and Activity Impairment Questionnaire:Ulcerative Colitis.

Bowel Urgency Remission was associated with significant decrease in all 4 scores at Week 12 (*P* = .0018 for absenteeism; *P* < .0001 for the remaining scores; Table 2) and Week 52 (*P* = .0203 for absenteeism; *P* < .0001 for the remaining scores; Table 2).

#### Fatigue

Bowel Urgency CMI and Bowel Urgency Remission were associated with significant reduction in Fatigue NRS scores at Week 12 and Week 52 (*P* < .0001; Table 2).

#### Abdominal Pain

Patients who achieved Bowel Urgency CMI or Bowel Urgency Remission showed significant reduction in Abdominal Pain NRS scores at Week 12 and Week 52 (*P* < .0001; Table 2).

#### Patient Global Rating of Severity

**Figure 4.**
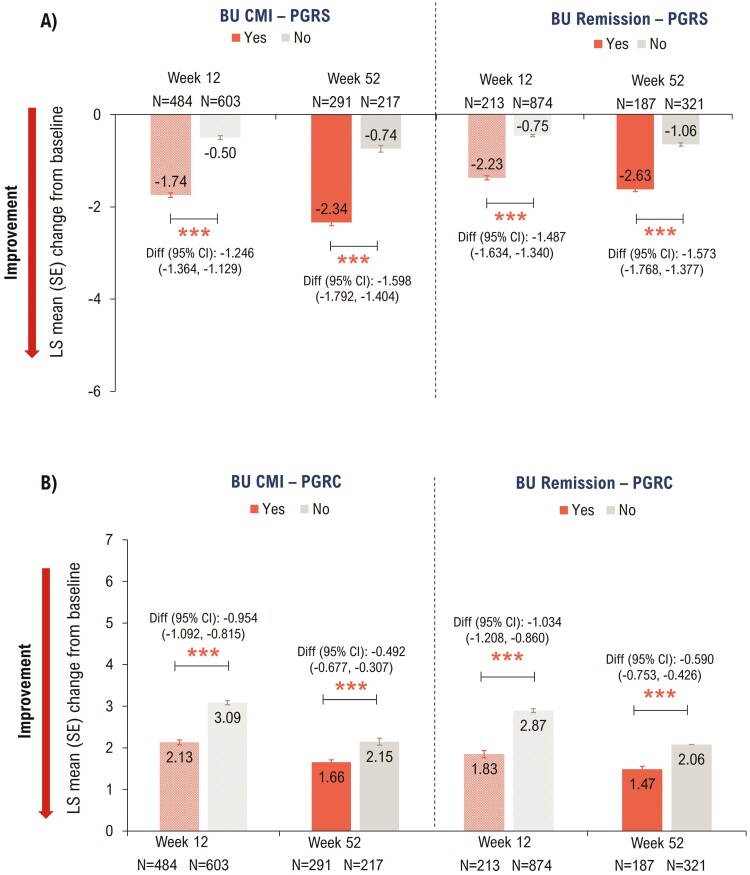
Association between Bowel Urgency CMI or Remission and PGRS and PGRC at Weeks 12 and 52—mITT with baseline Urgency NRS ≥3^a^. *P*-values were calculated using ANCOVA with mBOCF for missing data imputation. ***P < .0001 for the association of PGRS and PGRC outcomes with Bowel Urgency CMI or Remission (Yes vs No). ^a^Induction study analyses included mITT patients with baseline Urgency NRS ≥3. Maintenance study analyses included patients who had Urgency NRS ≥3 at baseline and achieved clinical response with mirikizumab at Week 12 (LUCENT-1). ANCOVA, analysis of covariance; BU, bowel urgency; CI, confidence interval; CMI, Clinically Meaningful Improvement; Diff, difference; LSM, least squares mean; mBOCF, modified baseline observation carried forward; mITT, modified intent-to-treat; NRS, Numeric Rating Scale; PGRC, Patient’s Global Rating of Change; PGRS, Patient’s Global Rating of Severity; SE, standard error.

Patients achieving Bowel Urgency CMI showed significant improvement in PGRS scores at Weeks 12 and 52 (*P* < .0001; Figure 4A). Similarly, patients who achieved Bowel Urgency Remission showed significant improvement in PGRS scores at Weeks 12 and 52 (*P* < .0001; Figure 4A).

#### Patient Global Rating of Change

Patients with Bowel Urgency CMI achieved significant improvement in PGRC scores at Weeks 12 and 52 (*P* < .0001; Figure 4B). Likewise, patients with Bowel Urgency Remission showed significant improvement in PGRC scores at Week 12 and Week 52 (*P* < .0001; Figure 4B).

#### Nocturnal stool

**Figure 5.**
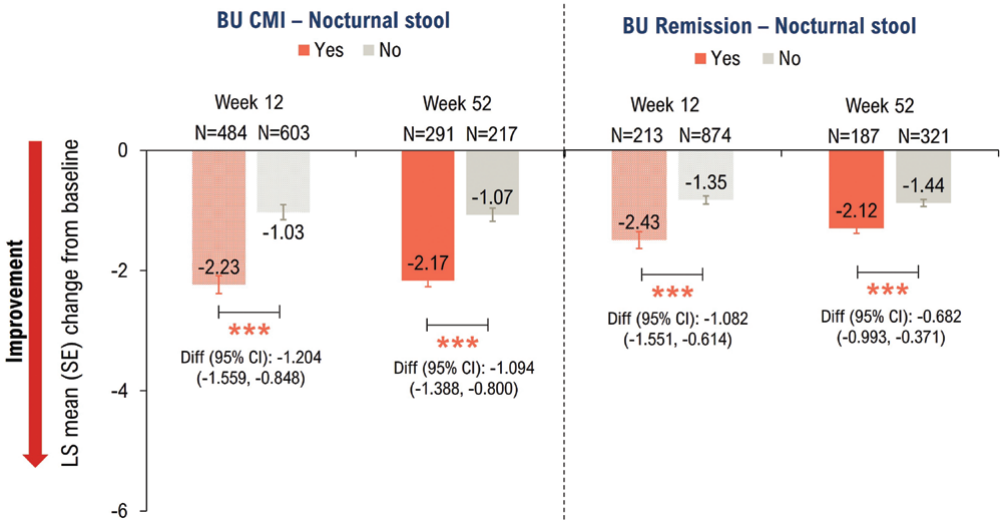
Association between Bowel Urgency CMI or Remission and Nocturnal Stool at Weeks 12 and 52 − mITT with baseline Urgency NRS ≥3^a^. *P*-values were calculated using ANCOVA with mBOCF for missing data imputation. ***P < .0001 for the association of Nocturnal Stool with Bowel Urgency CMI or Remission (Yes vs No); analysis includes mITT population with a Nocturnal Stool score ≥1 at baseline. ^a^Induction study analyses included mITT patients with baseline Urgency NRS ≥3. Maintenance study analyses included patients who had Urgency NRS ≥3 at baseline and achieved clinical response with mirikizumab at Week 12 (LUCENT-1). ANCOVA, analysis of covariance; BU, bowel urgency; CI, confidence interval; CMI, Clinically Meaningful Improvement; Diff, difference; LSM, least squares mean; mBOCF, modified baseline observation carried forward; mITT, modified intent-to-treat; NRS, Numeric Rating Scale; SE, standard error.

Patients who achieved Bowel Urgency CMI or Bowel Urgency Remission showed significant decrease in Nocturnal Stool scores at Weeks 12 and 52 (*P* < .0001; Figure 5).

## Discussion

In this analysis of patients with moderately-to-severely active UC from LUCENT-1 and LUCENT-2 phase 3 studies, we observed that Bowel Urgency CMI or Remission is associated with improvement in both disease-specific (IBDQ and WPAI:UC) and general (SF-36 and EQ-5D-5L) QoL outcomes as well as symptom defined PROs (Fatigue NRS, Abdominal Pain NRS, PGRS, PGRC, and Nocturnal Stool). The improvements in these outcomes were significantly higher in patients who achieved Bowel Urgency CMI or Remission, compared to those who did not. For both Bowel Urgency CMI and Bowel Urgency Remission, the association of bowel urgency improvement with QoL and PRO assessments was strong at Week 12 and was sustained through Week 52 for all outcomes.

In agreement with Urgency NRS qualitative study data,^21^ bowel urgency, was identified as an important symptom to patients with UC in independently developed PROs such as Symptoms and Impacts Questionnaire for UC,^34^ UC-PRO Signs and Symptoms,^35^ and UC-Symptom Questionnaire.^36^ These PROs assess bowel urgency along with measurement of SF and RB. The Urgency NRS was developed in accordance with the Food and Drug Administration guidance for new PROs.^37^ Since bowel urgency is a subjective symptom and experience may differ across patients, a NRS is considered appropriate to assess urgency severity in UC.^21^ While protected by copyrights it is freely available as a tool for patient studies.

Across the IBDQ domains, Bowel Urgency CMI or Bowel Urgency Remission was associated with QoL improvement at Weeks 12 and 52. These results corroborate previous findings that assessed the impact of bowel urgency on QoL of patients with moderately-to-severely active UC.^10,18^ Absence of bowel urgency was strongly associated with IBDQ improvement after adjusting for SF and RB, suggesting an independent association between bowel urgency and QoL.^10^ Additionally, an online survey of Japanese patients with UC confirmed that urgent defecation correlates with a decrease in the patient’s QoL.^8^ Another cross-sectional study of 743 patients with UC demonstrated that bowel urgency decreased QoL, as measured by SF-36, after adjusting for treatment, age, and clinical manifestations.^9^

The current data expand on the known relationship of bowel urgency to QoL outcomes by examining several disease-specific and general QoL instruments and assessing the relationship between bowel urgency and PRO measures using a well-defined patient population from the LUCENT-1 and LUCENT-2 clinical trials.

In addition to the direct impact on patients’ QoL and activities, bowel urgency has also been associated with clinical outcomes. A cross-sectional and subsequent longitudinal study within IBD Partners, a patient-powered research network, demonstrated that bowel urgency is independently associated with compromised QoL, future risk of hospitalization, corticosteroid use, and colectomy.^38^ In the phase 2 study of mirikizumab in UC, the absence of bowel urgency was associated with improved clinical measures of UC disease activity.^10^ Likewise, in the phase 3 LUCENT induction and maintenance studies, Bowel Urgency CMI or Remission were associated with better clinical outcomes, including clinical remission, corticosteroid-free remission, endoscopic remission, and symptomatic remission.^18^ These findings are in agreement with data from the phase 2 study of upadacitinib, where improvement in bowel urgency, measured by the presence or absence of the symptom, correlated with improvement in most clinical outcomes.^17^

Despite the importance of bowel urgency in patients with UC, previous survey-based studies have observed substantial variations between patients’ and physicians’ perceptions of the impact of these symptoms on patients’ lives.^39^ Only 38% of patients felt completely comfortable reporting bowel urgency to their healthcare providers. Of patients not comfortable reporting bowel urgency, 62% mentioned being embarrassed talking about it. Among healthcare providers, 75.5% reported they proactively discussed bowel urgency at routine appointments. Those healthcare providers who reported that they do not proactively discuss bowel urgency (24.5%) cited the main reason as they expect the patient to bring it up (46.9%).^40,41^

As bowel urgency is a subjective symptom, additional research is needed to characterize the clinical interpretation of individual scores on the Urgency NRS. In LUCENT-1 and LUCENT-2 trials, RB remission and SF remission rates were significantly higher in patients who achieved Bowel Urgency CMI or Remission versus those who did not (*P* < .001).^42^ In a phase 2 study in patients with moderate-to-severe UC, absence of urgency was associated with higher rates of RB remission, SF remission, and endoscopic healing (all *P* < .001), compared to patients who reported presence of urgency.^10^ These results suggest that bowel urgency could be used as a complementary marker of UC disease activity. In addition, results from mediation analyses^11-15^ and a phase 2 study^10^ support that bowel urgency is independently associated with improvement in clinical outcomes and QoL in patients with ulcerative colitis. Therefore, the current analyses were not adjusted for potential confounding effects of improvement in RB and SF.

Bowel urgency is an important and burdensome symptom for patients with UC. For patients with moderately-to-severely active UC, Bowel Urgency CMI or Remission is associated with improvement in QoL outcomes and PRO measures at Week 12. The association was sustained through Week 52. The study findings provide evidence for the impact of bowel urgency on QoL, which may help facilitate clinical decision making in patients with moderately-to-severely active UC and result in improved patient health and well-being.

## Supplementary Material

otae001_suppl_Supplementary_Tables_S1_Figures_S1-S2

## Data Availability

Lilly provides access to all individual participant data collected during the trial, after anonymization, with the exception of pharmacokinetic or genetic data. Data are available by request 6 months after the indication studied has been approved in the US and EU and after primary publication acceptance, whichever is later. No expiration date of data requests is currently set once data are made available. Access is provided after a proposal has been approved by an independent review committee identified for this purpose and after receipt of a signed data sharing agreement. Data and documents, including the study protocol, statistical analysis plan, clinical study report, blank or annotated case report forms, will be provided in a secure data sharing environment. For details on submitting a request, see the instructions provided at www.vivli.org
