# Supplementary material for: Association of Bowel Urgency With Quality-of-Life Measures in Patients With Moderately-to-Severely Active Ulcerative Colitis: Results From Phase 3 LUCENT-1 (Induction) and LUCENT-2 (Maintenance) Studies
Source: Crohns Colitis 360. 2024 Jan 6;6(1):otae001. doi: 10.1093/crocol/otae001 (PMC10838132; doi:10.1093/crocol/otae001)
Supplement: otae001_suppl_Supplementary_Tables_S1_Figures_S1-S2 [file otae001_suppl_supplementary_tables_s1_figures_s1-s2.docx]

**Association of bowel urgency with quality-of-life measures in patients with moderately-to-severely active ulcerative colitis: Results from phase 3 LUCENT-1 (induction) and LUCENT-2 (maintenance) studies**

**Supplementary Table 1: Detailed description of patient-reported QoL outcomes**

| **Patient-reported QoL measures** | |
| --- | --- |
| IBDQ ^1,2^ | The IBDQ questionnaire contains 32 items distributed across 4 domains/subscores: i) bowel symptoms, ii) systemic symptoms, iii) emotional function, and iv) social function. The items are graded on a 7-point Likert scale (7=“not a problem at all” and 1=“a very severe problem”) over a recall period of last 2 weeks. Total score (32–224) is the sum of the 4 domains/subscores. Higher score indicates better QoL. |
| SF-36 Version 2 ^3,4^ | The 36-item questionnaire, measures 2 overall summary scores (range: 0–100; PCS and MCS) and 8 health domain scores (range: 0–100; physical functioning, role-physical, role-emotional, bodily pain, vitality, social functioning, mental health, and general health) over a recall period of last 4 weeks. Higher scores indicate better function and/or health. |
| EQ-5D-5L VAS ^5,6^ | The questionnaire assesses patients’ current health status using visual analogue scale (VAS; range: 0 = “worst imaginable health state” to 100 = “best imaginable health state”). Higher score indicates better health state. |
| WPAI:UC ^7,8^ | WPAI:UC measures the impact of UC on work productivity and regular activities during the past 7 days. Four scores (absenteeism, presenteeism, activity impairment, and overall work impairment) are calculated as impairment percentages based on response to 6 items: i] employment status; ii] hours missed from work due to the disease, iii] and for hours missed from work due to other reasons; iv] hours actually worked; v] productivity affected by the disease while working, vi] productivity affected by the disease while performing regular unpaid activities. Patients who are employed complete the absenteeism, presenteeism, and overall work impairment questions, and all patients complete the activity impairment question. Higher scores indicate greater impairment and less productivity. |

IBDQ, Inflammatory Bowel Disease Questionnaire; MCS, Mental Component Summary; PCS, Physical Component Summary; QoL, quality of life; SF-36, Medical Outcomes Study 36-Item Short Form Health Survey; UC, ulcerative colitis; VAS, visual analog scale; WPAI:UC, Work Productivity and Activity Impairment Questionnaire:Ulcerative Colitis.

**Supplementary Figure 1: Association between Bowel Urgency CMI or Remission and IBDQ response (≥16-point improvement from baseline) and IBDQ remission (IBDQ score ≥170) rates at Week 12 and Week 52 − mITT with baseline Urgency NRS ≥3^a^**

**
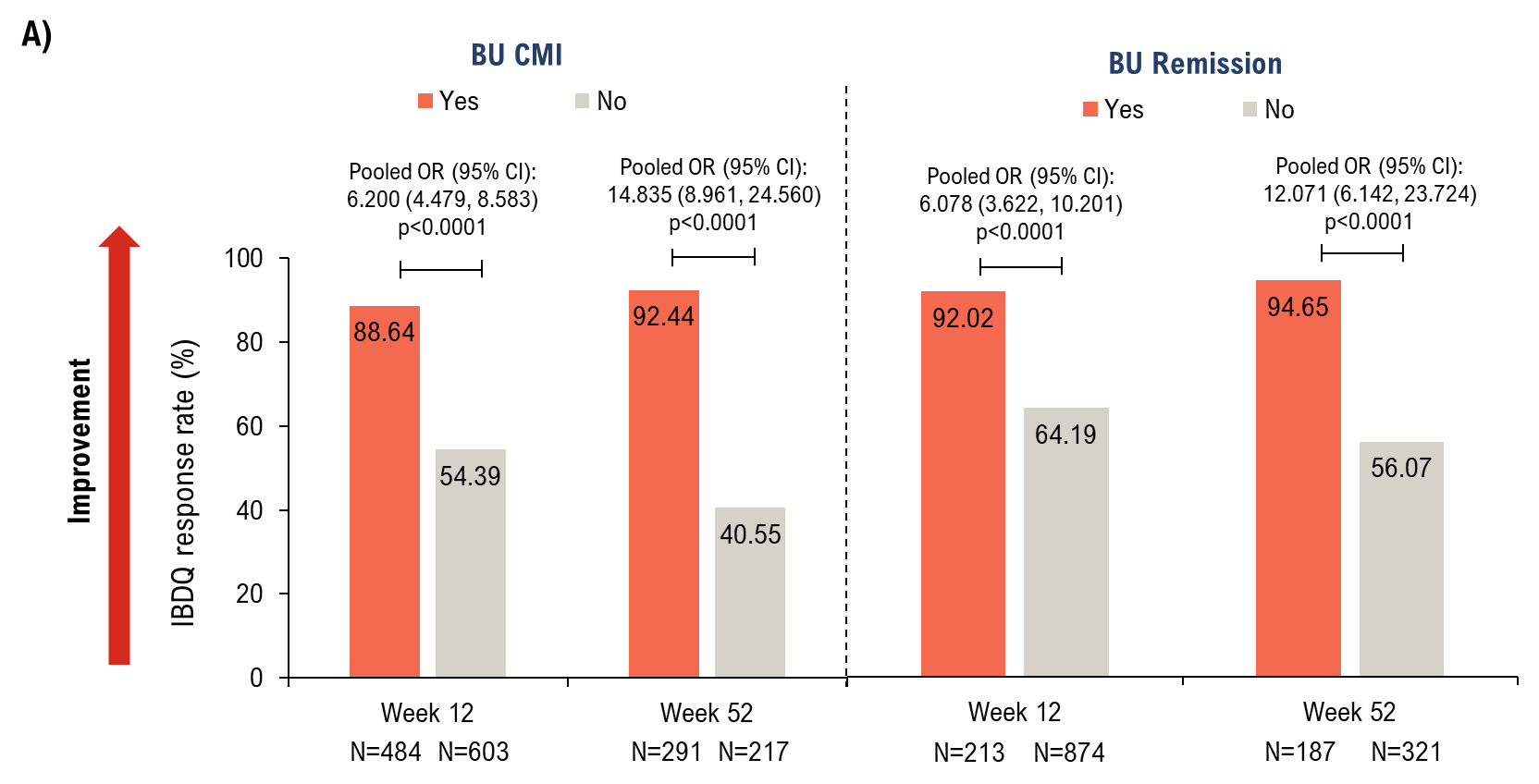
**

**
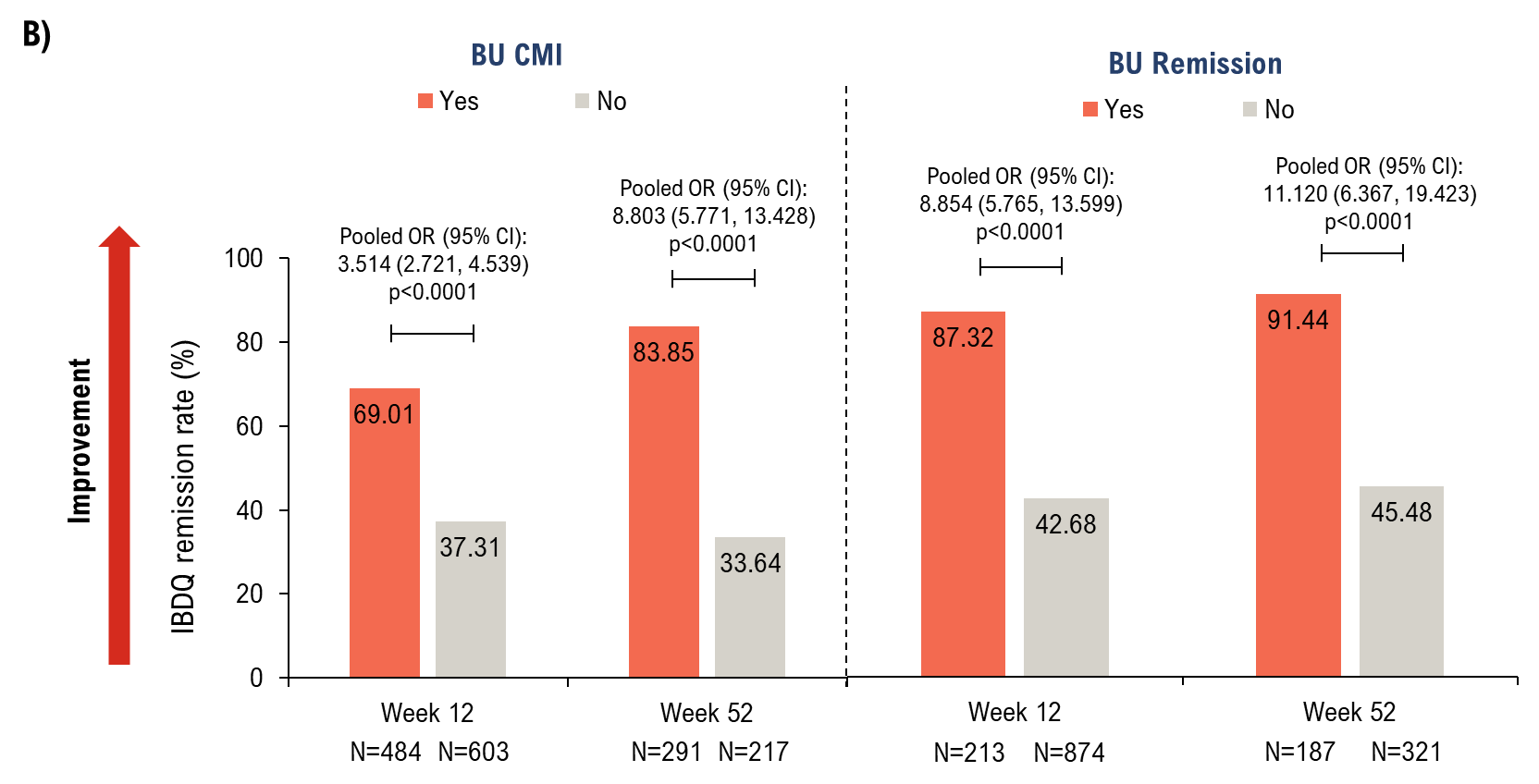
**

Data are presented as proportion of patients who achieved MCID response using CMH with NRI.

^a^Induction study analyses included mITT patients with baseline Urgency NRS ≥3. Maintenance study analyses included patients who had Urgency NRS ≥3 at baseline and achieved clinical response with mirikizumab at Week 12 (LUCENT-1).
BU, bowel urgency; CI, confidence interval; CMI, Clinically Meaningful Improvement; CMH, Cochran-Mantel-Haenszel; IBDQ, Inflammatory Bowel Disease Questionnaire; OR, odds ratio; mITT, modified intent-to-treat; NRI, non-responder imputation; NRS, Numeric Rating Scale.

**Supplementary Figure 2: Association between Bowel Urgency CMI or Remission and SF-36 PCS and MCS MCID (≥5-point improvement from baseline) response rates at Week 12 and Week 52 − mITT with baseline Urgency NRS ≥3^a^**

**
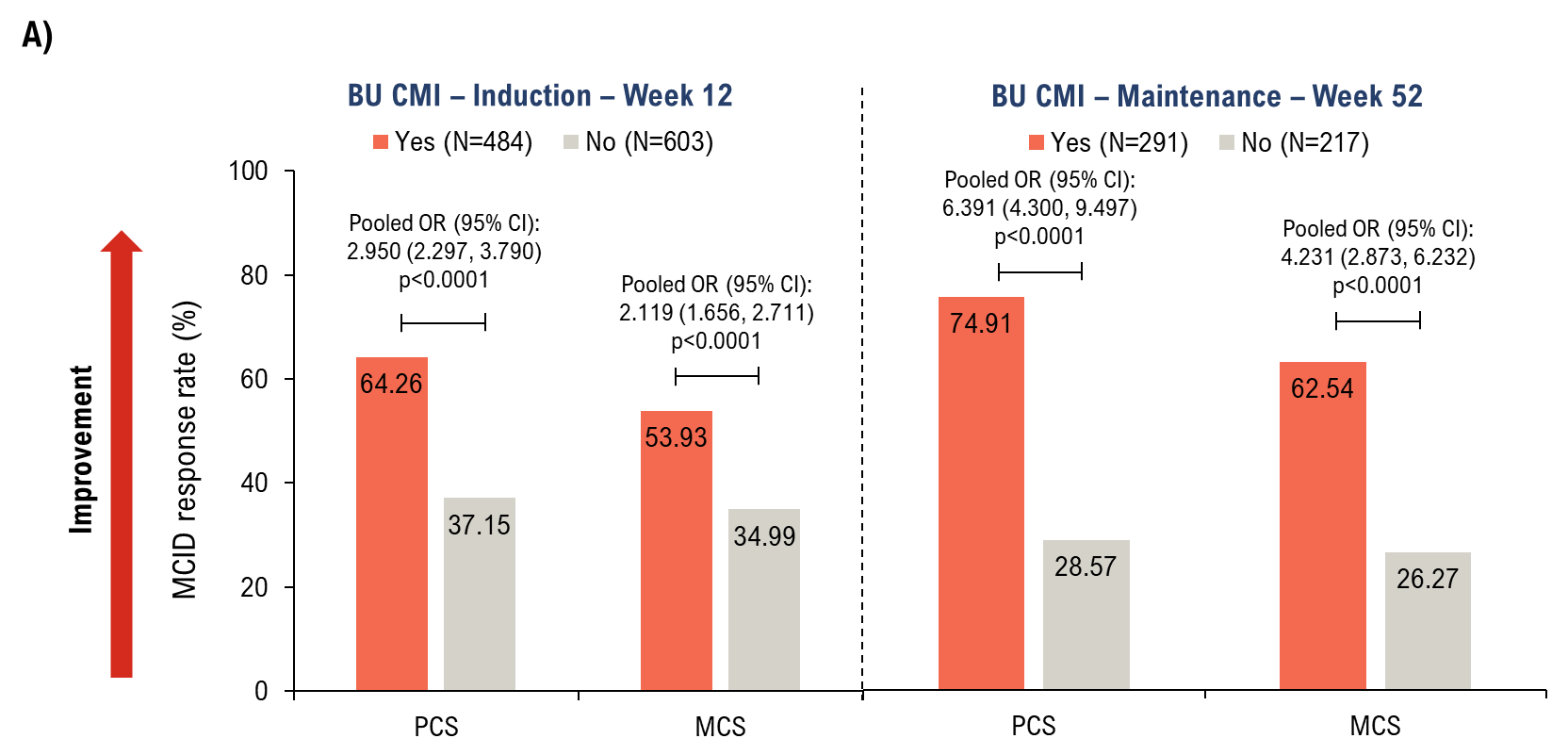
**

**
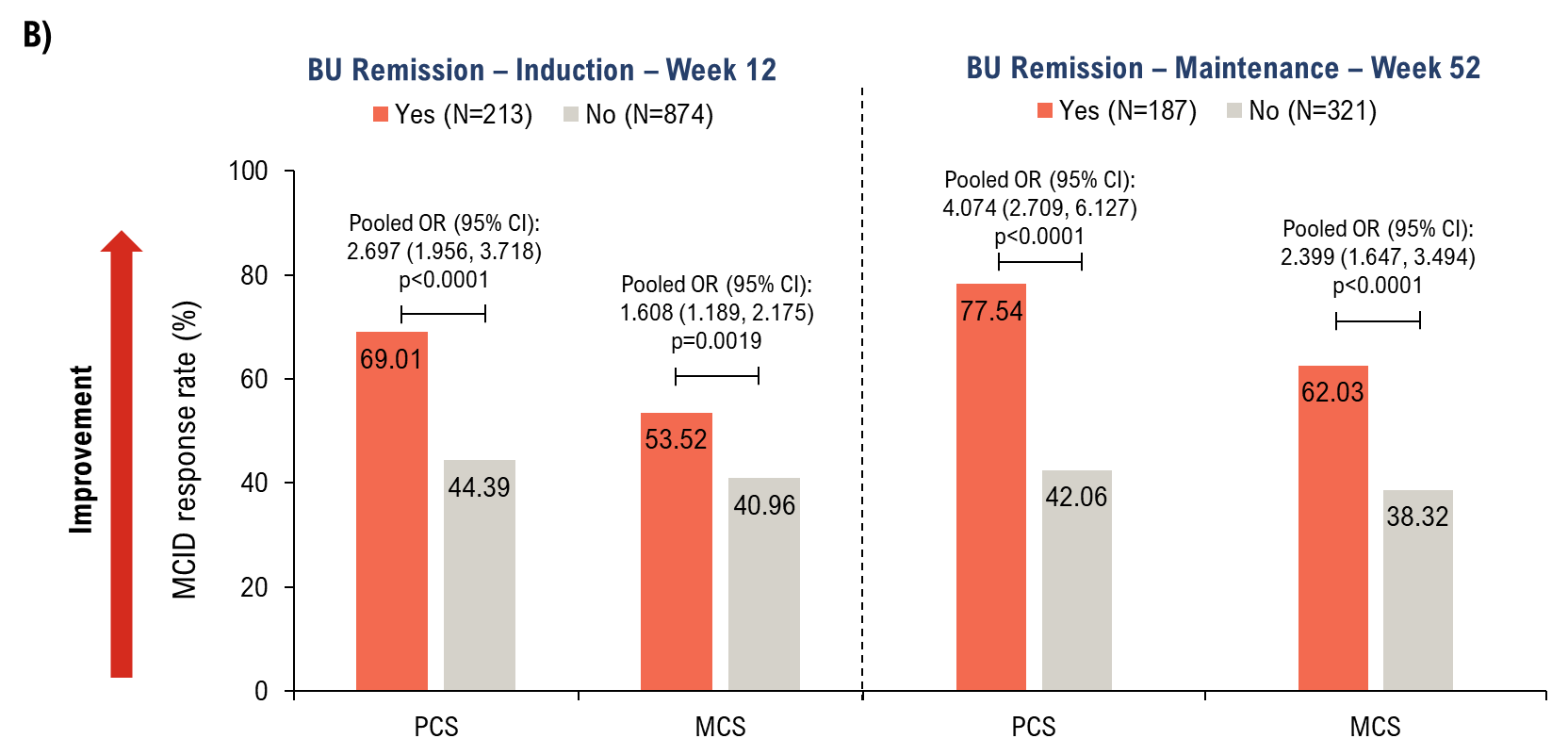
**

Data are presented as proportion of patients who achieved MCID response using CMH with NRI.

^a^Induction study analyses included mITT patients with baseline Urgency NRS ≥3. Maintenance study analyses included patients who had Urgency NRS ≥3 at baseline and achieved clinical response with mirikizumab at Week 12 (LUCENT-1).
BU, bowel urgency; CI, confidence interval; CMI, Clinically Meaningful Improvement; CMH, Cochran-Mantel-Haenszel; OR, odds ratio; MCID, minimal clinically important difference; MCS, Mental Component Summary; mITT, modified intent-to-treat; NRI, non-responder imputation; NRS, Numeric Rating Scale; PCS, Physical Component Summary; SF-36, Medical Outcomes Study 36-Item Short Form Health Survey.

**References**

1. Irvine EJ, Zhou Q, Thompson AK. The short inflammatory bowel disease questionnaire: A quality of life instrument for community physicians managing inflammatory bowel disease. CCRPT investigators. Canadian Crohn's relapse prevention trial. Am J Gastroenterol 1996;91:1571–8.

2. Guyatt G, Mitchell A, Irvine EJ*, et al.* A new measure of health status for clinical trials in inflammatory bowel disease. Gastroenterology 1989;96:804–10.

3. Ware JE, Jr., Sherbourne CD. The MOS 36-item short-form health survey (SF-36). I. Conceptual framework and item selection. Med Care 1992;30:473–83.

4. Maruish M, Kosinski M, et al. User’s manual for the SF-36v2 health survey. 3rd ed. Lincoln, (Rhode Island): QualityMetric Incorporated; 2011.

5. Herdman M, Gudex C, Lloyd A*, et al.* Development and preliminary testing of the new five-level version of EQ-5D (EQ-5D-5L). Qual Life Res 2011;20:1727–36.

6. EuroQol research foundation. EQ-5D-5L user guide, 2019. Available from: <Https://euroqol.Org/publications/user-guides>. Accessed 12 July 2022.

7. [Reilly associates] Reilly associates WPAI scoring. Available at: <Http://www.Reillyassociates.Net/wpai_scoring.Html>. Accessed 12 July 2018.

8. Reilly MC, Zbrozek AS, Dukes EM. The validity and reproducibility of a work productivity and activity impairment instrument. Pharmacoeconomics 1993;4:353–65.
